# Supermicrosurgical Suture-Stent Technique for A Lymphaticovenular Bypass

**DOI:** 10.3390/jcm10122595

**Published:** 2021-06-11

**Authors:** Ryo Karakawa, Hidehiko Yoshimatsu, Keisuke Kamiya, Yuma Fuse, Tomoyuki Yano

**Affiliations:** Department of Plastic and Reconstructive Surgery, Cancer Institute Hospital of the Japanese Foundation for Cancer Research, 3-8-31 Ariake, Koto-ku, Tokyo 135-8550, Japan; hidehiko.yoshimatsu@gmail.com (H.Y.); keisuke.kamiya@jfcr.or.jp (K.K.); yuma.fuse@jfcr.or.jp (Y.F.); tomoyuki.yano@jfcr.or.jp (T.Y.)

**Keywords:** lymphedema, lymphaticovenular anastomosis, suture-stent technique, ICG lymphography

## Abstract

Background: Lymphaticovenular anastomosis (LVA) is a challenging procedure and requires a sophisticated supermicrosurgical technique. The aim of this study was to evaluate and establish a discrete supermicrosurgical anastomosis method using the “suture-stent technique”. Methods: Forty-eight LVA sites of twenty patients with lower extremity lymphedema who had undergone LVA between July 2020 and January 2021 were included in this study. LVA was performed with the conventional technique or with the suture-stent technique. The patency of the anastomoses was evaluated using an infrared camera system intraoperatively. The success rate on the first try and the final success rate for each group were compared. Results: After full application of the exclusion criteria, 35 LVAs of 16 patients including 20 limbs were included in the analysis. The ratio of good patency findings after anastomosis in the suture-stent technique group was 100%. The incidences of leakage or occlusion on the first try were statistically greater in the conventional technique group (29.4%) than in the suture-stent technique group (0%) (*p* = 0.0191). All anastomoses achieved good patency in the final results. Conclusion: With its minimal risk of catching the back wall during the anastomosis, the suture-stent technique can be considered an optimal anastomosis option for LVA.

## 1. Introduction

Lymphedema is a debilitating condition caused by a failure of the lymphatic drainage system, and it is characterized by swelling and fibroadipose tissue deposition in the extremities [1,2]. Since the introduction of microsurgical lymphovenous anastomosis (LVA) by O’Brien et al. as one of the treatment modalities for lymphedema in 1977, which is aimed at symptom relief by creation of a bypass between the congested lymphatic vessel and vein, there have been multiple refinements [3]. After the introduction of the supermicrosurgical technique for LVA, which uses lymphatics and venules smaller than 0.8 mm, by Koshima et al., many studies have reported the successful outcome of performing LVA in lymphedema patients with various modifications [4,5,6,7,8,9,10]. However, LVA is still a challenging procedure for microsurgeons and requires a sophisticated supermicrosurgical technique because thin-walled lymphatic vessels, whose diameter is usually smaller than 0.8 mm, are difficult to detect and anastomose [11]. Inserting forceps into small lumens is difficult, and the back wall of a vessel can easily be inadvertently caught during anastomosis.

The aim of this study was to evaluate and establish a discrete supermicrosurgical anastomosis technique using the “suture-stent technique” in LVA. To appreciate the reliability of the suture-stent technique, a comparison was made between this technique and the conventional technique.

## 2. Materials and Methods

Institutional Review Board approval was obtained. We retrospectively identified patients with lymphedema who underwent LVA by one surgeon (the first author) from July 2020 to January 2021 at our hospitals. We excluded the patients with iodine allergy. We excluded LVA sites in which intraoperative indocyanine green (ICG) lymphography showed no light enhancement before the anastomosis or where end-to-side anastomosis had been performed. Finally, 35 LVA sites of 16 patients were included in this study.

Collected clinical and intraoperative findings included sex, age, body mass index, duration of edema, radiotherapy history, cellulitis history, clinical stages based on the International Society of Lymphology (ISL) classification, anatomical location of the incision site, dermal backflow stage based on ICG lymphography, and lymphosclerosis severity of the lymphatic vessels. Clinical stages were classified into five categories based on the ISL classification: stage 0, stage I, stage IIa, stage IIb, stage III. The anatomical locations of the incision sites were classified into the following three anatomical locations: the groin, the thigh, and the lower leg. Dermal backflow stages based on ICG lymphography were classified into six stages: stage 0, only a linear pattern was observed; stage I, a linear pattern and a splash pattern were observed; stage II, a linear pattern and one region with a stardust pattern were observed; stage III, a linear pattern and two regions with a stardust pattern were observed; stage IV, a linear pattern and three regions with a stardust pattern were observed; stage V, a stardust and/or diffuse pattern were observed [12]. The lymphosclerosis severity of the lymphatic vessels was classified into four categories based on the NECST classification: Normal, Ectasis, Contraction, and Sclerosis [13].

### 2.1. Surgical Technique

Preoperative ICG lymphography was performed as reported previously. First, 0.2 mL of ICG (Diagnogreen 0.25%; Daiichi Sankyo Pharmaceuticals, Tokyo, Japan) was injected subcutaneously into the bilateral lower extremities of the first and fourth web spaces of the foot and into the lateral and medial malleolus [12,14]. After the ICG injection, circumferential fluorescent images of lymphatic drainage channels were obtained using an infrared camera system (Photodynamic Eye; Hamamatsu Photonics K.K., Hamamatsu, Japan). LVA was planned in the site with a linear pattern, just distal to the beginning of the dermal backflow region. The subcutaneous vein was detected near the planned area using ultrasonography [15].

**Figure 1:**
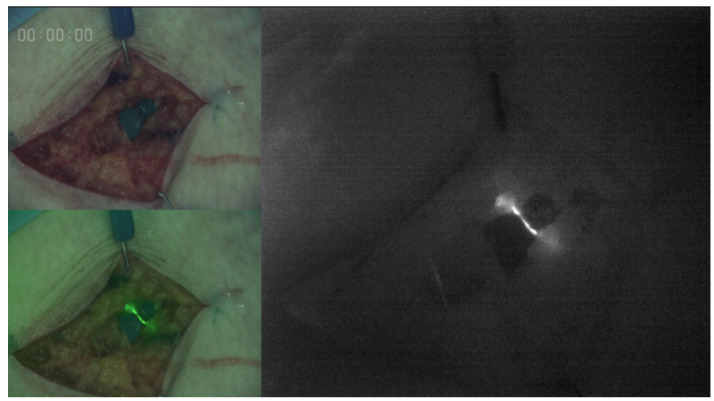
The lymphatic vessel was identified using an infrared camera system (LIGHTVISION; Shimazu Corporation, Kyoto, Japan) before the anastomosis.

After the skin incision, the lymphatic vessel was identified using an infrared camera system (Photodynamic Eye; Hamamatsu Photonics K.K., Hamamatsu, Japan, LIGHTVISION; Shimazu Corporation, Kyoto, Japan), and was anastomosed to the vein using 12-0 nylon (Figure 1).

The anastomoses were performed with either the conventional technique or the suture-stent technique. After confirmation of the patency of the anastomosis site, the incision site was closed.

### 2.2. Details of the Anastomosis Technique

#### 2.2.1. Conventional Technique

**Figure 2:**
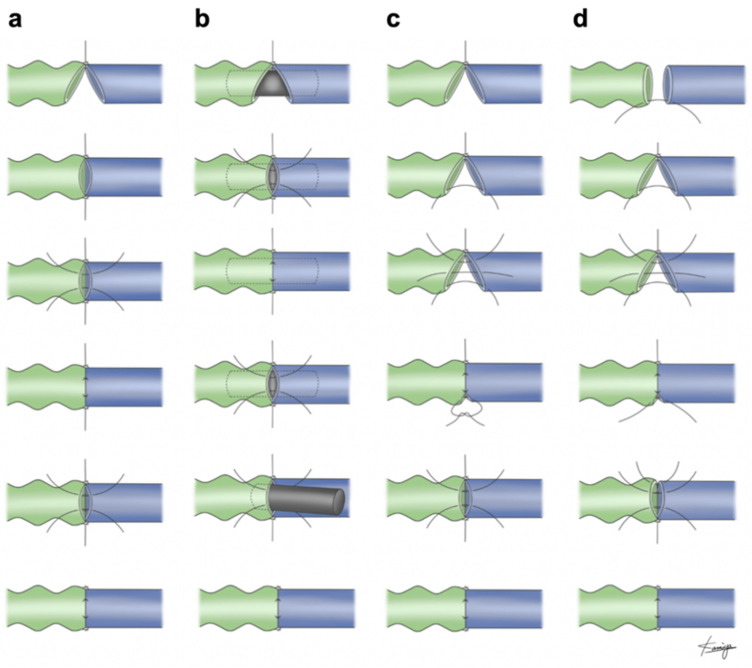
Schema of lymphaticovenular anastomosis: (**a**) the conventional technique, (**b**) the intravascular stent (IVaS) technique, (**c**) the open guide suture technique, (**d**) the suture-stent technique.

The first two sutures were placed and tied horizontally 180° apart. Two or three interrupted sutures were placed on the anterior wall with the untied method and tied. Then the vessels were turned over and two or three interrupted sutures were placed on the posterior wall with the untied method and tied (Figure 2a) [16].

#### 2.2.2. Suture-Stent Technique

**Figure 3:**
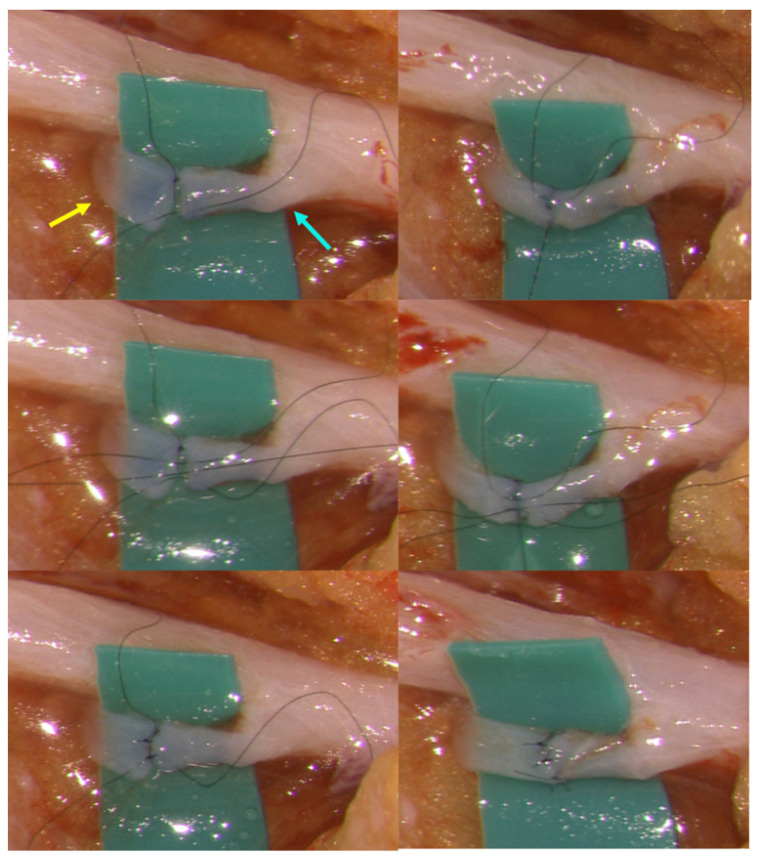
Intraoperative view of lymphaticovenular anastomosis (LVA) with the suture-stent technique: yellow arrow—lymphatic vessel; blue arrow—vein.

The first stay suture was placed, and the second stay suture was placed horizontally 180° apart from the first stay suture. The second stay suture was tied, and the first stay suture was left untied. After two or three interrupted sutures were placed on the anterior wall with the untied method and tied in order, the vessels were turned over. Two or three interrupted sutures were placed on the posterior wall and tied in order. Then the first stay suture was tied at the end of the anastomosis. (Figure 2d and Figure 3, Supplementary Video S1) All the sutures were inserted from the lumen to the outside of the lymphatic vessels.

### 2.3. Outcome Evaluation

**Figure 4:**
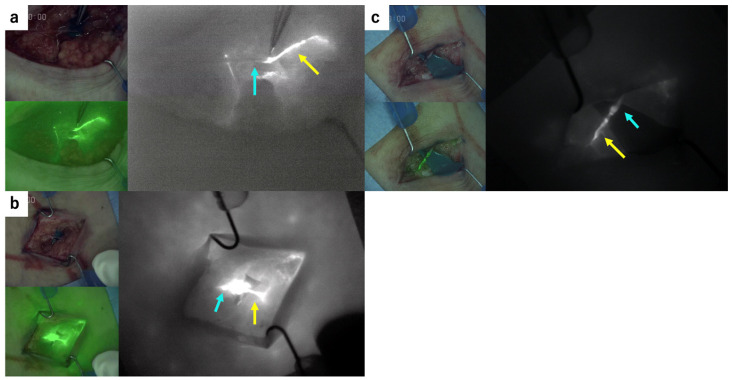
The evaluation of patency of the anastomoses using an infrared camera system intraoperatively: (**a**) occlusive finding, (**b**) leaky finding, (**c**) good patency. yellow arrow: lymphatic vessel, blue arrow: vein.

The patency of the anastomoses was evaluated by the surgeon using an infrared camera system intraoperatively and the ICG findings were categorized into three types: good patency, occlusive, and leaky. (Figure 4) The anastomosis site was taken down and re-anastomosed when the occlusive findings were detected. Additional sutures were added when leaky findings were detected. After re-anastomosis or the suture addition, the patency of the anastomosis was evaluated again. The success rate on the first try and the final success rate were evaluated in both the conventional technique group and the suture-stent technique group.

### 2.4. Statistical Analysis

Descriptive statistics were obtained. Student’s t-test was used to compare the diameter of the lymphatic vessels and veins in the conventional technique group and the suture-stent technique group. Fisher’s exact test was used to compare the anatomical location of the incision site, the ISL classification, the dermal backflow stage based on ICG lymphography, and the lymphosclerosis severity of the lymphatic vessels in the conventional technique group and the suture-stent technique group. All statistical tests were two-sided, and a value of *p* < 0.05 was considered to be statistically significant. All statistical analysis was performed using R v. 4.0.2 (R Foundation for Statistical Computing, Vienna, Austria).

## 3. Results

### 3.1. Patient Characteristics

Sixteen patients and twenty limbs were included in this study. The mean age was 63.2 years (range 33–91; SD 16.0). There were 5 males (31.2%) and 11 females (68.7%). The mean body mass index (BMI) was 25.0 (range 17.6–35.4; SD 5.6). The causative disease of lymphedema was cervical cancer in four patients, uterine cancer in two patients, ovarian cancer in three patients, other malignancy in two patients, and primary lymphedema in five patients. The mean number of anastomoses was 2.6 (range 1–4; SD 0.9). The mean operative time was 162 min (range 47–300; SD 65.0). Lymphedema severity was at stage I in four limbs, stage IIa in six limbs, stage IIb in six limbs, and stage III in four limbs based on the ISL classification. Dermal backflow stage was at stage I in one limb, stage II in 12 limbs, and stage III in seven limbs. Demographics are listed in Table 1.

### 3.2. Anastomosis Characteristics

Among the 16 participants enrolled in this study, 44 LVAs were performed. LVAs were excluded if intraoperative ICG lymphography showed no light emission before the anastomosis (*n* = 8) or if end-to-side anastomosis was performed (*n* = 1), resulting in 35 LVAs being included in the analyses. Of 35 LVAs, the conventional technique was used in 17 anastomoses and the suture-stent technique was used in 18 anastomoses. Comparing the conventional technique group and the suture-stent technique group, there were no statistically significant differences in the anatomical location of the incision site, the ISL classification, the dermal backflow stage based on ICG lymphography, the NECST classification, the mean diameter of lymphatic vessels, or the mean diameter of veins: (*p* = 0.725, *p* = 0.176, *p* = 0.218, *p* = 0.855, *p* = 0.925, and *p* = 0.933, respectively) (Table 2).

### 3.3. ICG Findings After Anastomosis

The ratio of good patency findings after anastomosis in the suture-stent technique group was 100%. The incidences of leakage or occlusion on the first try were statistically greater in the conventional technique group than in the suture-stent technique group. (*p* = 0.0191) All anastomoses achieved good patency in the final results. Therefore, there were no statistical differences in the final results between the conventional technique group and the suture-stent technique group (*p* > 0.99) (Table 3).

## 4. Discussion

In the setting of the microvascular anastomosis, various methods have been reported as alternatives to the conventional microsurgical anastomosis technique [16,17,18,19]. Since the introduction of the supermicrosurgery technique by Koshima et al. in the 1990s [20], several studies have reported on the supermicrosurgical anastomosis technique which can be applied to vessels smaller than 0.8 mm [16,17,18,19]. Ozkan et al. introduced the open guide suture technique in 2005 [17] (Figure 2c). The difference with the conventional technique is the order of suture-tying. Since the second stay suture is left untied, the view of the lumen is greatly improved in this technique. Narushima et al. applied the temporary intravascular stenting (IVaS) technique for supermicrosurgical anastomosis in 2008 [16] (Figure 2b). The IVaS technique is used to perform anastomosis of very small vessels when forceps cannot be inserted into the lumen. In this method, a nylon stent is placed into the lumen to eliminate the risk of catching the posterior wall. In 2009, a comparative study of the conventional technique, the IVaS technique, and the open guide suture technique for microsurgical anastomosis using vessels of rats was conducted by Miyamoto et al. [16]. They recommended the open guide suture technique as a new standard for the anastomosis of very small vessels because it provides a clear view of the lumen and simplified anastomosis.

In the setting of the LVA, microsurgeons require a more sophisticated technique than when performing anastomosis of small arteries or veins because of the peculiarities of lymphatic vessels; a lymphatic vessel has a thinner, transparent wall and its back wall can easily become inadvertently caught during anastomosis, and it is very difficult to see the lumen of lymphatic vessels of lymphedema patients, even under a microscope. Narushima et al. applied the IVaS technique to LVA and indicated the usefulness of this method [21]. However, this method has several drawbacks—the insertion and removal of the stent are cumbersome, require a certain amount of experience, and carry the risk of damaging the vascular intima. Thus, we applied the modified open guide suture technique, named the “suture-stent technique”, to LVA.

The suture-stent technique of LVA confers several advantages. First, the view of the lumen of the lymphatic vessel and vein is clearer than with the conventional technique because the first stay suture is left untied. In our technique, all the sutures were inserted from the lumen to the outside of the lymphatic vessels. This reduces the risk of catching the back wall of the lymphatic vessel during the anastomosis. Second, this technique does not require any additional devices such as an intravascular stent, which eliminates the need for a complicated procedure and the risk of damaging the intima of vessels. Third, an appropriate number of needles can be inserted to prevent leakage after the anastomosis even if there is a caliber difference. In our study, the suture-stent technique group showed no leaky or occlusive findings on the first try. Moreover, the incidences of leakage or occlusion on the first try were significantly greater in the conventional technique group than in the suture-stent technique group. This confirmed that the suture-stent technique is a useful method for obtaining good patency in LVA.

There are several differences between the open guide suture technique and our suture-stent technique. First, the view of the lumen of the lymphatic vessel is clearer when inserting the second suture because the first stay suture is left untied. Second, the view of the lumen of the lymphatic vessel is clearer and there is a lower risk of catching the anterior wall when inserting the fifth and sixth sutures into the posterior wall because the first stay suture is left untied (Figure 2). Our method is advantageous in that it minimizes the risk of catching the back wall during the anastomosis.

One of the drawbacks of this technique is that the stay sutures can come out during the anastomosis because many stay sutures are left untied. Another drawback is that the thread may become stuck because there are many remnant sutures until the last tie. However, microsurgeons can get used to coping with this issue as they follow the learning curve.

Confirmation of the patency of the anastomosis with the ICG tracing inside the vein is extremely important to obtain a good result [22]. In our study, the patency of all the anastomoses was evaluated using an infrared camera system intraoperatively. Additional procedures such as re-anastomosis or suture additions were performed when occlusive or leaky findings were detected. Ultimately, good patency findings were achieved in all LVAs.

Of 44 LVAs enrolled in our study, eight (18.1%) lymph-collecting vessels showed no ICG enhancement and were excluded from our study. Johnson et al. reported that 36.1% of all lymphatic collecting vessels were non-ICG enhanced in their LVA cases, and concluded that non-ICG enhanced lymphatic collecting vessels should also be considered as functional if they have lymphatic flow [23]. Therefore, non-ICG enhanced lymphatic vessels should be anastomosed more carefully because the anastomosis cannot be evaluated using an infrared camera system intraoperatively.

The greatest limitation of this study is that operator-dependent variables have to be considered because the anastomosis method was chosen depending on one surgeon’s preference. Nonetheless, our results showed a high success rate for the suture-stent technique group. However, we believe this could be justified because the purpose of this study is to present the reliability of the suture-stent technique, not to demonstrate its superiority over the conventional technique.

## 5. Conclusions

With its minimal risk of catching the back wall during the anastomosis, the suture-stent technique can be considered an optimal anastomosis option for LVA.

## Figures and Tables

**Table 1:** Patient demographics.

Parameter		Value
No. of patients (no. of limbs)		16 (20)
Age (years)	Average (range)	63.2 (33–91)
Sex	Female (%)	11 (68.7)
	Male (%)	5 (31.2)
BMI	Average (range)	25 (17.6–35.4)
No. of anastomosis	Average (range)	2.6 (1–4)
Operation time	Minutes (range)	162 (47–300)
Lymphedema duration (years)	Average (range)	3.6 (0.25–15)
Cause of disease	Cervical cancer (%)	4 (25)
	Uterine cancer (%)	2 (12.5)
	Ovarian cancer (%)	3 (18.7)
	Other malignancy (%)	2 (12.5)
	Primary lymphedema (%)	5 (31.2)
Radiation therapy	Positive (%)	1 (6.2)
	Negative (%)	15 (93.7)
History of cellulitis	Positive (%)	9 (56.2)
	Negative (%)	7 (43.7)
ISL stage	I (%)	4 (20)
	IIa (%)	6 (30)
	IIb (%)	6 (30)
	III (%)	4 (20)
DB stage	I (%)	1 (5)
	II (%)	12 (60)
	III (%)	7 (35)

BMI: body mass index; ISL: International Society of Lymphology classification; DB: dermal backflow.

**Table 2:** Comparison of the anastomosis demographics between the conventional technique group and the suture-stent technique group.

Characteristic		Conventional Technique (*n* = 17)	Suture-Stent Technique (*n* = 18)	*p*
Location	Groin (%)	1	0	0.725
	Thigh (%)	5	7	
	Lower leg (%)	11	11	
ISL stage	I (%)	4	2	0.176
	IIa (%)	2	8	
	Iib (%)	7	6	
	III (%)	4	2	
DB stage	I (%)	0	1	0.218
	II (%)	9	13	
	III (%)	8	4	
NECST	Normal	12	15	0.855
	Ectasis	2	2	
	Sclerosis	1	3	
Mean diameter	Lymphatic vessel (mm)	0.54	0.53	0.925
	Vein (mm)	0.69	0.68	0.933

ISL: International Society of Lymphology classification, DB: dermal backflow, NECST: Normal, Ectasis, Contraction, and Sclerosis Type.

**Table 3:** Comparison of the anastomosis ICG findings after anastomosis between the conventional technique group and the suture-stent technique group.

Characteristic		Conventional Technique (*n* = 17)	Suture-Stent Technique (*n* = 18)	*p*
First try	Good patency (%)	12 (70.5)	18 (100)	0.0191
	Leakage (%)	2 (11.7)	0 (0)	
	Occlusion (%)	3 (17.6)	0 (0)	
Final result	Good patency (%)	17 (100)	18 (100)	≥0.999

ICG: indocyanine green.

## Data Availability

The data presented in this study are available on request from the corresponding author.

## Supplementary Materials

The following are available online at https://www.mdpi.com/article/10.3390/jcm10122595/s1, Video S1: Suture-stent technique.
